# Practical approach to diagnose and manage benign liver masses

**DOI:** 10.1097/HC9.0000000000000560

**Published:** 2024-10-30

**Authors:** Reshma Reguram, Aishwarya Ghonge, Justin Tse, Renumathy Dhanasekaran

**Affiliations:** 1 Division of Gastroenterology and Hepatology, Department of Medicine, Stanford University, Stanford, California, USA; 2 Department of Radiology, Stanford University, Stanford, California, USA

**Keywords:** adenoma, cyst, FNH, hemangioma

## Abstract

Benign liver lesions are among the most commonly diagnosed abnormalities in liver imaging. They are often discovered incidentally during routine examinations or imaging conducted for unrelated reasons. These can be solid lesions, such as hemangiomas, focal nodular hyperplasia, hepatic adenomas, or cystic lesions. Recent advancements in MRI technology, particularly with hepatocyte-specific contrast agents, have enhanced the characterization of these lesions, reducing the reliance on invasive tissue sampling. Nevertheless, tissue sampling retains a crucial role in the evaluation of indeterminate lesions or those with malignant potential. While most benign liver lesions are asymptomatic, some can become symptomatic, causing discomfort, pain, or bleeding, particularly if the lesion is large. A deep understanding of the molecular underpinnings of the lesions is crucial for tailoring patient management strategies, particularly in distinguishing lesions that require surgical intervention from those that can be monitored. For instance, the molecular subclassification of hepatic adenomas has provided mechanistic insights and identified certain subtypes that are at higher risk of malignancy. Most benign liver lesions can be safely monitored; however, in patients with cirrhosis or a known primary malignancy, a high index of suspicion for cancer is required. It is crucial to carefully evaluate any liver lesion identified in these patients to ensure that indeterminate lesions are not overlooked. Effective management of benign liver lesions involves a multidisciplinary team, including hepatologists, surgeons, and radiologists, ensuring a comprehensive and individualized approach to patient care. This review outlines the clinical presentation of common benign liver lesions, providing a diagnostic and management framework. Emphasis is placed on a personalized approach to minimize patient distress and optimize outcomes by leveraging imaging advancements and multidisciplinary collaboration.

## INTRODUCTION

Benign liver lesions are among the most commonly diagnosed abnormalities in liver imaging,^1^,^2^ often discovered incidentally during routine examinations or imaging conducted for unrelated indications.^3^,^4^,^5^ These lesions are typically asymptomatic and include a spectrum of diagnoses, such as simple cysts, hemangiomas, focal nodular hyperplasia (FNH), hepatic adenomas, and others. Although liver lesions are often benign, their detection can cause significant anxiety and distress for patients as they navigate the complex diagnostic process. Thus, it is crucial for clinicians to possess the skills necessary to diagnose and manage benign liver lesions, ensuring they can provide reassurance and effective care to their patients.

The widespread adoption of advanced cross-sectional imaging techniques has markedly increased the detection rates of incidental liver lesions.^6^ Imaging modalities such as ultrasound, CT, and MRI are now commonplace in clinical practice, revealing incidental liver lesions in patients who are often asymptomatic. Importantly, the majority of these patients do not have underlying cirrhosis or chronic liver disease, and liver enzymes are typically normal, which further complicates the diagnostic pathway. Recent advancements in MRI techniques have revolutionized the diagnostic approach to benign liver lesions.^7^,^8^ Contrast-resolution imaging with hepatocyte-specific agents can provide detailed characterization of these lesions,^9^,^10^ reducing the uncertainties in diagnosis. This noninvasive diagnostic tool allows for a more precise evaluation of the nature of the liver lesion, thereby decreasing the reliance on tissue sampling. Nevertheless, tissue sampling retains a critical role, particularly for indeterminate lesions where imaging alone is inconclusive, and it remains the cornerstone in diagnosing cases where malignancy cannot be confidently excluded from imaging.

While generally benign, these liver lesions can occasionally lead to clinical symptoms. Complications such as abdominal discomfort, pain, or, more rarely, internal bleeding may occur, particularly with larger lesions or those located in areas that can lead to compression of surrounding tissues. Furthermore, certain hepatic adenoma subtypes harbor a small risk of malignant transformation, which necessitates careful monitoring and, sometimes, surgical intervention.^11^,^12^ The management of patients with benign liver lesions benefits immensely from a multidisciplinary approach involving hepatologists, surgeons, and radiologists. This collaborative framework ensures comprehensive assessment and tailored management, balancing the need for intervention against the potential risks of invasive procedures.

As benign liver lesions continue to be identified with increasing frequency, the importance of distinguishing between those requiring intervention and those that can be safely monitored cannot be overstated. Ongoing research involves the development of predictive algorithms to better identify lesions at risk of complications or malignancy and innovations in imaging that could further reduce the need for invasive tests. Ultimately, the goal is to refine the accuracy of diagnosis and optimize the management of these benign liver lesions, thereby minimizing patient distress and improving outcomes. In this review, we will describe the epidemiology and clinical presentation of the common benign lesions and provide a practical diagnostic and management framework, emphasizing the importance of a personalized approach.

## HEPATIC HEMANGIOMAS

### Epidemiology and clinical features of hepatic hemangiomas

Hepatic hemangiomas are the most common solid benign lesions in the liver.^1^ They contain clusters of blood-filled cavities lined by a single layer of endothelial cells and are fed by the hepatic arterial circulation. They have a prevalence of 3%–20%, with the highest incidence rate in adults between 30 and 50 years.^13^ Women are more commonly affected with a female-to-male ratio of 4.5:1 to 5:1. Most hemangiomas are <5 cm in diameter, and they usually remain stable, asymptomatic, and without complications.^14^,^15^ These hemangiomas are most commonly found in the right lobe of the liver.^16^ The etiology of the disease could potentially be genetic in a subset of patients. External factors such as hormonal exposure, specifically steroid therapy, estrogen therapy, and pregnancy, have also been associated with increased risk of hepatic hemangioma growth and can increase the risk of bleeding and rupture.^13^ The exact mechanisms by which hormones impact hemangiomas remain unclear, given that these tumors do not express estrogen or progesterone receptors. Thus, oral contraceptives, hormone replacement therapy, or anabolic steroids are not contraindicated in patients with hemangiomas.^17^ At diagnosis, most hemangiomas are asymptomatic. However, giant hemangiomas can cause symptoms like abdominal discomfort, nausea, early satiety and rarely jaundice, or high output cardiac failure.^16^ The definition of a giant hemangioma varies, with some experts considering them giant if they exceed 5 cm, while others reserve this classification for those over 10 cm.^18^ We generally align with the latter definition, yet it is important to recognize that this issue is a continuum, where the location and symptoms of the hemangioma often hold more significance than just the size alone. Very rarely, giant hemangiomas can lead to a clinical presentation of Kasabach-Merritt syndrome, the constellation of which includes hemolytic anemia, thrombocytopenia, prolonged prothrombin time, and hypofibrinogenemia.^19^,^20^ This is more common in children than adults.

### Diagnostic strategies for hepatic hemangiomas

Hepatic hemangiomas are highly prevalent, which makes it extremely important to distinguish them from other hypervascular liver lesions which may be malignant, such as HCC and metastatic cancer. The characteristic imaging features of hemangiomas across various modalities, primarily ultrasound, CT, and MRI, allow for a noninvasive and accurate diagnosis in most cases. The diagnostic approach for suspected hemangiomas typically starts with ultrasound, followed by CT or MRI for definitive characterization. On ultrasound, they appear homogeneously hyperechoic with posterior acoustic enhancement. Grayscale ultrasound has 94% sensitivity and 80% specificity in differentiating hepatic hemangiomas from hyperechoic malignant lesions that are <3 cm.^13^,^21^ However, it has an imperfect specificity because other lesions, such as HCC, may also be hyperechoic; thus, diagnosing a hepatic hemangioma exclusively with grayscale ultrasound should be made with caution and only in an otherwise healthy patient without a known history of malignancy or chronic liver disease.^22^ Color Doppler does not show intratumoral vessels due to very slow intralesional flow, but contrast-enhanced ultrasound (CEUS) does show the classic enhancement pattern of hemangiomas seen on CT and MRI, which includes peripheral, nodular, and discontinuous enhancement and progressive fill-in over time.^23^ At multiphasic CT, hemangiomas may show characteristic enhancement patterns as described above for CEUS, with the addition that the enhancement follows the blood pool (ie, aorta).^24^ This enhancement pattern reflects the blood pooling within the vascular spaces of the hemangioma. By the delayed phases, the lesion may become completely filled with contrast and hyperdense compared to the surrounding liver tissue, which is known as “fill-in.” Unenhanced CT may show a circumscribed, hypoattenuating lesion but would require contrast for further characterization.

**FIGURE 1:**
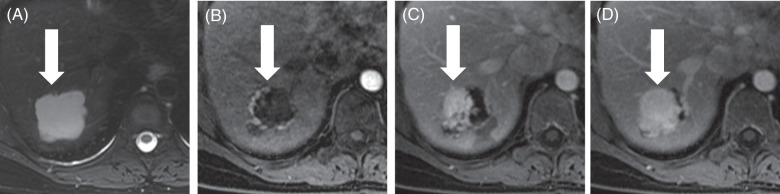
Hepatic hemangioma—Characteristic features on MRI with contrast. A 53-year-old woman with a hepatic hemangioma (arrow) undergoes MRI with extracellular contrast. (A) Axial T2-weighted images show a lobulated, circumscribed, and moderately T2-hyperintense lesion. (B) After injection of contrast, axial T1-weighted images in the arterial phase show that the lesion has peripheral, nodular, and discontinuous areas of enhancement. The signal intensity of these enhancing areas is similar to that of the aorta, that is, blood pool. (C) In the portal venous and (D) delayed phases, the areas of enhancement increase centripetally and continue to match the blood pool.

MRI is particularly effective for diagnosing hemangiomas as it incorporates additional sequences, including T2-weighted images, and has additional postcontrast phases as it does not involve ionizing radiation (Figure 1). On MRI, hemangiomas are typically moderately hyperintense (bright) on T2-weighted images—a hallmark of these lesions.^25^ This higher signal intensity on T2-weighted images is due to the slow flow of blood through the cavernous vascular spaces of the hemangioma. During dynamic contrast-enhanced MRI studies, hemangiomas show a similar enhancement pattern to multiphasic CT, beginning with peripheral, nodular, and discontinuous enhancement and progressive fill-in over time with signal intensity following the aorta. Importantly, the contrast media used in MRI, particularly extracellular gadolinium-based agents, provides excellent delineation of these dynamic imaging features.

A small proportion of hemangiomas can have atypical imaging features. An important subset is the sclerosing hemangioma. As the name suggests, these lesions are characterized by collagenous matrix deposition, usually in the center, and hemangiomas may undergo sclerosis in the setting of long-standing chronic liver disease. Such hemangiomas may be ambiguous after initial imaging with MRI. Nevertheless, it is important to distinguish these lesions from other potentially malignant lesions that may appear similarly in patients with chronic liver disease, such as an intrahepatic cholangiocarcinoma. The diagnosis can be confidently made if remote imaging shows the presence of a definite hemangioma in the same location in which serial examinations showed sclerosis over time. In the absence of remote imaging, a suspected sclerosed hemangioma can be evaluated with technetium-99m–labeled red blood cell scan, positron emission tomography/CT, or may require further evaluation through a biopsy. While biopsies carry a theoretical risk for complications like bleeding, the actual rates are low in clinical practice, and thus suspected hemangiomas may be biopsied safely if necessary.^26^ This procedure provides definitive histological information, which is crucial for the accurate diagnosis and management of lesions where noninvasive methods fail to provide clear results. Other rare variants of hemangioma include flash-filling hemangiomas, calcified hemangiomas, and multifocal hemangiomas also known as hemangiomatosis. Most of these can be diagnosed on contrast-enhanced MRI.

Hemangiomas require more meticulous evaluation in 2 clinical contexts: when they occur against a background of fatty liver disease or cirrhosis. Hepatic steatosis, especially when severe, can affect the typical ultrasound appearance of hemangioma, making them appear hypoechoic with a perilesional halo.^27^ In the context of cirrhosis, hemangiomas are extremely rare as they undergo sclerosis, and when they do occur, the enhancement patterns can be challenging to distinguish from primary liver malignancies.^28^ Hemangiomas should be diagnosed with extreme caution in patients with cirrhosis and only after other malignant processes with targetoid enhancement patterns are considered to be excluded. Comparison with prior imaging and the use of contrast MRI are particularly helpful in both scenarios to confirm the diagnosis of hemangioma.

### Practical approach to the management of hemangiomas

**FIGURE 2:**
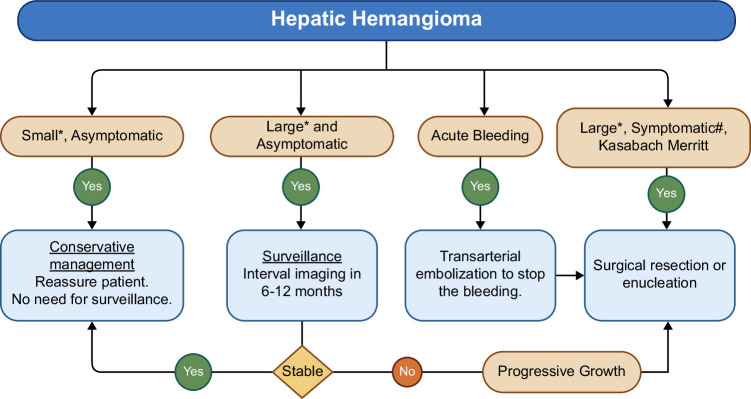
Practical approach to the management of hepatic hemangiomas. *Small—usually <5–7 cm; large—usually ≧5–7 cm. ^#^Symptomatic—Need to ensure that symptoms are plausibly related to hemangioma, and other causes are ruled out.

Figure 2 presents a practical approach to the management of hepatic hemangiomas. Most hepatic hemangiomas are small (<5 cm), asymptomatic, and stable in size, with patients generally having normal liver function. In such patients, if imaging shows characteristic features of hemangiomas, no further treatment or imaging surveillance is needed, and patients should be reassured of the lack of risk for malignant transformation. If the hemangioma is large (>5–7 cm) at diagnosis, it is reasonable to perform imaging surveillance for 6–12 months to ensure stability in size. More extended observation is necessary for individuals with symptoms clearly related to hemangioma, those on estrogen therapy, or during pregnancy to look for progressive growth.

For large lesions that continue to grow or directly cause symptoms, therapeutic interventions may be necessary. However, before considering interventions, it is essential to rule out other causes for the symptoms, such as gastroesophageal reflux disease, peptic ulcer disease, cholelithiasis, or irritable bowel syndrome. Surgical options like resection or enucleation are the cornerstone for the management of symptomatic hemangiomas or progressively enlarging giant hemangiomas.^29^ However, advancements in minimally invasive techniques have shifted preferences toward options such as transarterial embolization or ablative therapies. Surgery remains necessary for large or deeply embedded hemangiomas but carries small risks for hemorrhage or infection.^30^,^31^ Increasing use of minimally invasive laparoscopic techniques for both resection and enucleation is expected to further minimize surgical risks.^32^,^33^ Liver transplantation is very rarely employed for unresectable complicated hemangiomas, accounting for <0.02% of all liver transplants.^34^ Transcatheter arterial embolization is usually performed to control acute bleeding or reduce the size of the hemangioma before surgery. However, it can be used as the mainstay of therapy in patients who are suboptimal surgical candidates.^35^,^36^,^37^,^38^ Even if less invasive than surgery, transcatheter arterial embolization can still be complicated by an acute infarct, abscess, or biloma. Radiofrequency or microwave ablation can safely treat small hemangiomas; however, these small lesions can typically be monitored conservatively since they are unlikely to be the cause of the patient’s symptoms.

## FOCAL NODULAR HYPERPLASIA

### Epidemiology and clinical presentation of FNH

FNH is the second most frequent benign solid liver lesion and accounts for 8% of all primary liver tumors in clinical practice. Its overall prevalence is estimated to be 0.3%–3% in the general population,^17^ with the highest prevalence in 30–50-year-old women, with a female-to-male ratio of 6–8:1.^39^ There is no clear link between the development, size, or prognosis of FNH and hormonal exposure from taking oral contraceptive pills (OCPs) or pregnancy.^40^,^41^ This even raises the possibility that the prevalence of FNH in women is inflated because of higher likelihood of women getting imaging for pregnancy or other reasons. FNH are usually well-demarcated, nonencapsulated solid lesions composed of nodules of hyperplastic hepatocytes and expand as kupffer cells in the background of normal liver histology and are believed to develop in response to an aberrance in vasculature.^42^ FNH is usually asymptomatic and tends to be detected incidentally on imaging.^1^ Only one-third or fewer patients present with abdominal pain or discomfort. It can range anywhere between 2 cm and 20 cm in size, albeit up to 85% of FNH are <5 cm in size. While most FNHs remain stable in size, 10% can show growth, and another 10% can regress over time.^43^,^44^ FNH is thought to be at virtually no risk of undergoing malignant transformation, necrosis, rupture, or hemorrhage, and thus FNH usually does not need treatment unless it causes symptoms. In contrast, hepatic adenomas are more likely to bleed or rupture, thus requiring surveillance and interventions. This makes it crucial to accurately diagnose and differentiate between FNH and adenomas.

### Diagnostic strategies for FNH

FNH is usually discovered incidentally on imaging done for other reasons. It appears hypoechoic on grayscale ultrasound and often mimics other liver lesions, such as hepatic adenoma and even malignancy, which need vastly different approaches to management. For this reason, it is important to distinguish FNH from these lesions through imaging techniques that allow visualization of the arterial blood supply of the lesion. CEUS has advantages in that it enables real-time dynamic visualization of enhancement patterns of tumors.^45^ The central scar, if present, is not well-depicted by grayscale ultrasound, but visualization is improved by CEUS. On CEUS, FNH shows centrifugal vascularity, with non-rim arterial phase hyperenhancement and sustained portal venous and delayed phase enhancement.^46^ The central scar remains hypoechoic (nonenhancing) during the portal and late phases of CEUS.

**FIGURE 3:**
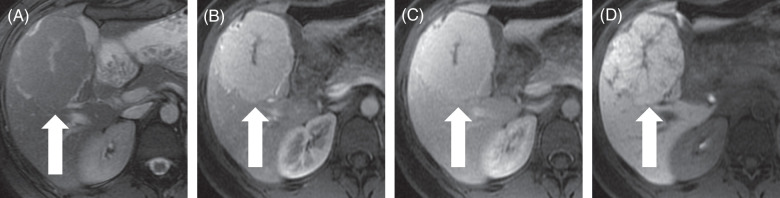
Focal nodular hyperplasia—Characteristic features on MRI with contrast. A 22-year-old woman with focal nodular hyperplasia (arrow) undergoes MRI with hepatobiliary contrast. (A) Axial T2-weighted images show a lesion with T2 iso- to minimal hyperintensity. (B) After injection of contrast, axial T1-weighted images in the arterial phase show that the lesion has homogeneous hyperenhancement with the exception of a central, stellate area known as the central scar. (C) In the portal venous phase, the enhancement persists slightly above the liver. (D) In the hepatobiliary phase, the mass is mostly iso- to hyperintense compared to the uninvolved liver; the central scar is most pronounced in this phase.

FNH are considered “stealth lesions” since they are iso- to slightly hypoattenuating on nonenhanced and portal venous phase CT scans and appear iso- to mildly T1-hypointense or iso- to mildly T2-hyperintense on MRI images.^47^ A central stellate scar is considered the hallmark imaging feature of FNH but is only present in 20% of cases and is usually seen in lesions >3 cm in size. Other distinguishing attributes are the vascular features of FNH, which are more specific than the scar used to make the diagnosis. This can be identified by the presence of a characteristic central spoke-wheel pattern, with branching arteries radiating peripherally from the feeding artery at the center of the lesion.^44^ There are multiple imaging techniques that may help in delineating the vascularity of indeterminate focal liver lesions. Contrast-enhanced MR with a hepatobiliary agent (such as gadobenate dimeglumine or gadoxetate disodium) is considered gold standard for characterizing focal liver lesions and has an optimal sensitivity (97%) and specificity (100%) for FNH (Figure 3).^48^ Hepatobiliary phase imaging which is typically performed at 20 minutes after the administration of gadoxetate disodium is specifically helpful in differentiating FNH from other lesions. Since FNH is a hyperplastic outgrowth of hepatocytes, it retains the expression of hepatocyte-specific membrane transport proteins, which enable the uptake and excretion of the hepatobiliary MRI contrast into the bile.^48^ Thus, FNH almost always shows hepatobiliary contrast uptake, thus appearing hyperintense compared to nontumoral liver in that phase.^49^ However, lesions of nonhepatocyte origin, such as hemangiomas or metastases, appear hypointense during the hepatobiliary phase imaging as they do not preserve the expression of hepatocyte-specific membrane transport proteins.^50^ Even lesions of hepatic origin, such as hepatic adenomas or HCC, usually appear hypointense during the hepatobiliary phase since they may not have functional hepatocytes. A caveat is that well-differentiated HCC or certain hepatic adenoma subtypes sometimes can express hepatocyte-specific membrane transport proteins and, therefore, can rarely be seen as iso- or even hyperintense on the hepatobiliary phase.^51^,^52^ Atypical imaging features such as the absence of arterial phase hyperenhancement, hypointensity on hepatobiliary phase, and presence of intralesional fat or calcification warrant consideration of biopsy to confirm the diagnosis and exclude other tumors that may occur in this same demographic population, such as hepatic adenomas.

### Practical approach to the management of FNH

**FIGURE 4:**
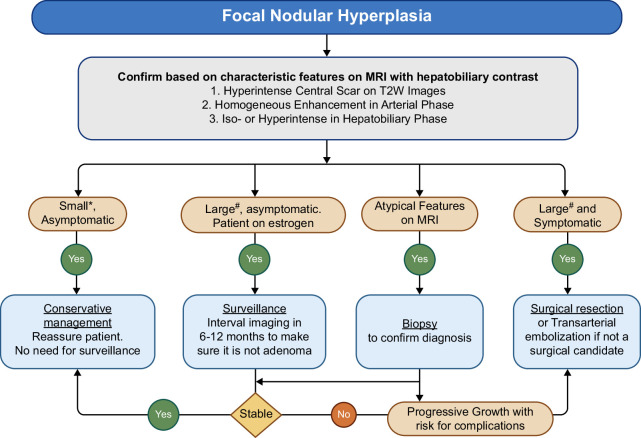
Practical approach to the management of focal nodular hyperplasia. *Small—usually <5–7 cm; ^#^Large—usually ≧5–7 cm; Symptomatic—Need to ensure that symptoms are plausibly related to hemangioma and other causes are ruled out.

Figure 4 presents a practical approach to the management of FNH. Management of FNH depends on whether the mass is symptomatic. It is useful to use hepatobiliary contrast-enhanced MRI to first confirm the diagnosis of FNH and exclude more sinister liver neoplasms. If lesions show atypical features on MRI, a biopsy can establish the diagnosis. If FNH is unequivocally confirmed on biopsy or imaging, no further surveillance imaging is needed. If imaging strongly suggests FNH but cannot conclusively rule out adenomas—especially in patients still using estrogen-containing pills—it is reasonable to conduct follow-up cross-sectional imaging in 6–12 months to verify the stability of the lesion size and confirm FNH before stopping surveillance. In rare instances, large FNH or progressively growing FNH can cause abdominal discomfort, and such patients may be considered for interventions either through elective surgery or through locoregional therapy such as transarterial embolization, stratified by risk for complications from surgery. If surgery is pursued, since FNH is not a premalignant lesion, a nonanatomic wedge resection or segmentectomy is performed, depending on the size of the lesion, to spare functional hepatic parenchyma. If surgical resection is not possible due to the size or location of the lesion, transarterial bland embolization can be offered.

## HEPATIC ADENOMAS

### Epidemiology and clinical presentation of hepatic adenomas

Hepatic adenomas are relatively rare benign liver tumors that predominantly affect women, with a female-to-male ratio of 9:1. These lesions were largely unknown before the introduction of estrogen-containing OCPs and anabolic steroids in the 1960s.^53^ The widespread adoption of oral contraceptives linked the increasing incidence of hepatic adenomas to the estrogen content of these pills. Although modern contraceptive pills contain much lower levels of estrogen compared to their first-generation counterparts, they remain a significant risk factor for hepatic adenomas. The annual incidence of adenomas in users of OCPs is ~30–40 cases per million, compared to 1 case per million among nonusers.^54^,^55^ Recent studies have shown an association between adenomas and metabolic disorders such as obesity and metabolic syndrome, indicating a shift in the etiology of these lesions.^56^,^57^,^58^ Other important associations that have been reported include anabolic steroids in men,^59^ and rare genetic syndromes such as glycogen storage disease,^60^ familial adenomatous polyposis,^61^ maturity-onset diabetes of the young,^62^ and McCune-Albright syndrome.^56^

Hepatic adenomas are classified into molecular subtypes, each with unique genetic, pathological, and radiologic characteristics that guide clinical management. The most common subtype is the inflammatory adenoma, which accounts for 30%–35% of hepatocellular adenomas.^23^,^63^ Apart from OCP use, these adenomas are associated with obesity, alcohol use, steatotic liver disease, and glycogen storage disorders. As a result of these coexisting factors, patients may present with elevated liver enzymes.^64^ Inflammatory adenomas carry mutations in *IL6ST*, *STAT3*, or *GNAS* and histologically show inflammatory infiltration, ductular reactions, sinusoidal dilatation, and dystrophic blood vessels, which are evident as pseudo portal tracks with thick-walled arteries but lacking veins or bile ducts. The second common subtype is the *HNF1A*-inactivated adenomas, which carry mutations in the transcription factor *HNF1A* ^57^,^63^ and are associated with MODY. Histopathologic analysis reveals macrovesicular fat and lack of liver fatty acid–binding protein expression. The third subtype of adenoma is the β-catenin–mutated variant, which carries a mutation in cadherin-associated protein β1 gene (*CTNNB1*) at exon 3 or exon 7/8.^63^ β-catenin–mutated adenomas at exon 3 have the highest risk for malignant transformation and are more common in men.^65^ β-catenin–mutated adenomas show atypical cells and aberrant nuclear expression of β-catenin and/or glutamine synthetase on immunohistochemistry. Apart from these classic subtypes, mixed variants can be seen, for example, the mixed inflammatory and β-catenin–mutated adenomas, which appear inflammatory on histology but also carry the *CTNNB1* mutation. Thus, all suspected adenomas should be evaluated with immunohistochemistry for β-catenin expression. A fourth, relatively new, and rare variant is the sonic hedgehog adenoma, characterized by activating mutations in this pathway and strongly associated with OCP use and obesity.^66^ These adenomas are highly vascular and prone to bleeding. Lastly, around 7% of adenomas do not fall into the above categories and remain unclassified.

hepatocellular adenomas often remain asymptomatic and are incidentally discovered during imaging studies for unrelated conditions. However, they may present with abdominal discomfort or pain, particularly if they are large or ruptured, leading to intra-abdominal hemorrhage. In general, the risk for bleeding increases with tumor size and reaches a significant level of >20% risk when adenomas are larger than 5cm.^67^,^68^ The β-catenin–mutated adenomas, sonic hedgehog adenomas, and inflammatory adenomas have the highest risk of bleeding, while HNF1A adenomas have the least. Another major dreaded complication of adenomas is the risk of malignant transformation to HCC. The major risk factors for HCC include β-catenin–mutated exon 3 subtype, tumor larger than 5 cm, male sex, and glycogen storage disorders.^63^ Most HCC that arises from hepatic adenomas appear to be well-differentiated with low risk for recurrence.^69^

### Diagnostic strategies for hepatic adenomas

**FIGURE 5:**
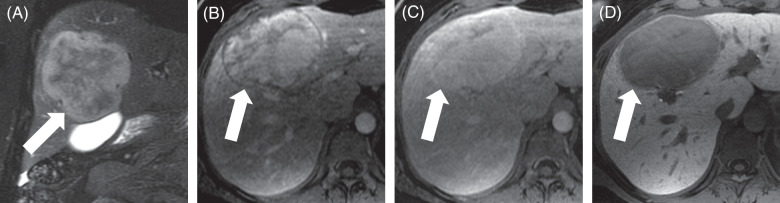
Hepatic adenomas—Characteristic features on MRI with contrast. A 35-year-old woman with metabolic syndrome and an inflammatory hepatocellular adenoma (arrow) undergoes MRI with hepatobiliary contrast. (A) Coronal T2-weighted images show a lesion with moderate T2 hyperintensity that is most pronounced at the periphery, also known as an atoll sign. (B) After injection of contrast, axial T1-weighted images in the arterial phase show that the lesion has heterogeneous hyperenhancement. (C) In the portal venous phase, the enhancement persists slightly above the liver. (D) In the hepatobiliary phase, the mass is hypointense compared to the uninvolved liver.

Hepatic adenomas generally do not have a characteristic appearance on ultrasound. Thus, MRI with contrast should be ordered whenever adenomas are suspected (Figure 5). MRI of the abdomen using gadoxetate disodium can potentially differentiate hepatic adenomas from FNH by focusing on the hepatobiliary phase, in which all subtypes of adenomas generally appear hypointense compared to the surrounding liver. However, it is important to note that a subset of adenomas, in particular atypical inflammatory adenomas or adenomas with β-catenin mutations, may be iso- or hyperintense in the hepatobiliary phase.^70^,^71^ Importantly, contrast-enhanced MRI can discern the specific subtype of hepatic adenoma in up to 80% of cases.^72^ We will describe some of the specific features associated with each subtype. *HNF1A-*mutated adenomas show the presence of diffuse, homogenous intralesional fat, which has a sensitivity of 95% and specificity of 85%.^73^ Inflammatory adenomas are moderately hyperintense on T2W images, and around a third of them have a peripheral rim of higher T2 signal intensity, called an atoll sign, since it resembles the shape of an atoll—a ring-shaped coral reef. This sign is indicative of sinusoidal dilatation and abnormal blood vessels in the periphery of these tumors and has a high specificity of 97% for the inflammatory subtype.^74^ The β-catenin–mutated exon 3 adenomas are usually iso-to hyperintense in the hepatobiliary phase or show a central scar.^75^ The β-catenin exon 7/8 adenomas and sonic hedgehog adenomas may not have specific imaging findings that help distinguish them, and their rarity makes it challenging to establish imaging patterns. Overall, advances in MRI contrast technology have significantly reduced the need for invasive tissue sampling by enabling precise visualization and differentiation of hepatic adenomas. However, if MRI is indeterminate for subtype identification or if the lesion exhibits atypical features that suggest malignancy, then tissue sampling is indicated.

### Practical approach to the management of hepatic adenomas

**FIGURE 6:**
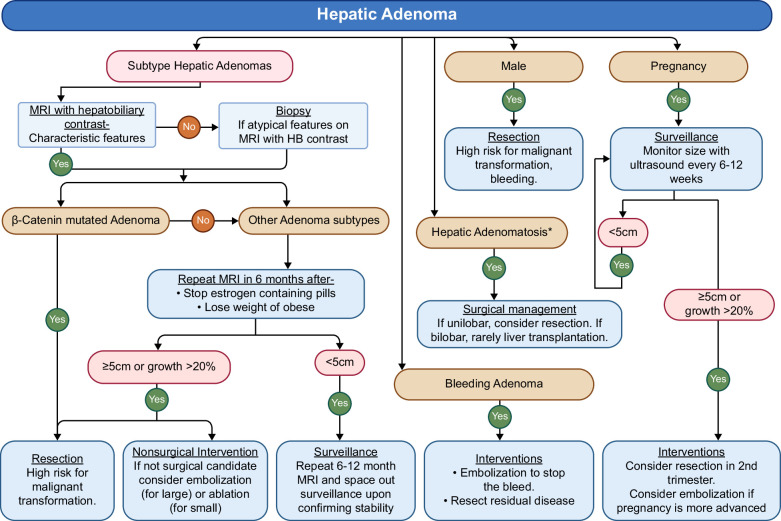
Practical approach to the management of hepatic adenomas.

Figure 6 presents a practical approach to the management of hepatic adenomas. The management of hepatocellular adenomas depends on several factors, including the size of the adenoma, the presence of symptoms, underlying risk factors, patient sex, and the anticipated risk of complications such as spontaneous bleeding or malignant transformation (Figure 2). For male patients, resection is recommended regardless of the size of the lesion due to the high risk of malignant transformation and bleeding. For all females, MRI with hepatobiliary contrast should be used to distinguish between the various subtypes. Resection is recommended in females if MRI or biopsy subtyping shows β-catenin–mutated subtype, if the adenoma is larger than 5 cm, or if it continues to grow more than 20% over 6 months despite risk factor modifications such as discontinuing estrogen-containing pills and/or weight loss. For adenomas <5 cm that remain stable or regress, MRI surveillance should be repeated after 1 year. If stability is confirmed, annual MRI scans are advised, with the option to space out surveillance over time. This approach mirrors general clinical practices as reflected in real-world studies, with most adenoma resections being carried out for lesions over 5 cm.^76^,^77^,^78^ In addition, in a large, multicenter study, the primary reasons for resection among lesions< 5 cm included suspicion of a premalignant lesion (55%), previous bleeding (14%), and male sex (11%).^76^

There are 4 special circumstances that dictate specific actions. First, adenomas presenting with bleeding require emergent attention and should be managed by hepatic artery embolization. If residual lesions are present in the 3–6-month surveillance imaging, it should be considered for resection. Second, if adenomas are diagnosed before or during pregnancy, they should be monitored with ultrasound every trimester. Resection or embolization may be considered in the second trimester if the adenoma is large or rapidly growing. Third, in cases of hepatic adenomatosis, especially in genetic conditions such as glycogen storage disorders in which patients have progressive chronic liver disease, liver transplantation can be considered. Fourth, if a high-risk adenoma is deemed unresectable due to technical difficulties or patient’s nonsuitability for surgery, embolization or ablation may be considered; however, patients will still require imaging surveillance. This approach ensures that the management of hepatic adenomas is tailored to individual patient characteristics and the specific nature of the adenoma, prioritizing both safety and efficacy in treatment planning.

## HEPATIC CYSTS

### Epidemiology and clinical presentation of hepatic cysts

Cystic lesions of the liver represent many varied disease processes, including but not limited to congenital malformations, inherited disorders, neoplasms, and infections.^79^ The female-to-male ratio is about 1.5:1 for asymptomatic simple cysts and 9:1 for symptomatic or complicated cysts, with large cysts almost exclusively found in women over 50 years old.^80^,^81^ Regardless of etiology, the overwhelming majority of the cysts are benign. The reported prevalence of hepatic cysts ranges from 3% to 18%, likely underestimating the true prevalence since these cysts are predominantly asymptomatic and typically discovered incidentally at imaging.

The most common cysts are simple hepatic cysts, which are fluid-filled masses lined by a single layer of epithelium of hepatic or biliary origin. They show a large variance in size, with the average being about 3 cm.^82^ There is no known correlation between simple cysts and estrogen exposure. They may present as a solitary mass or in multiples and are usually asymptomatic. However, simple cysts may become symptomatic due to increasing size or precarious location that leads to pressure symptoms. In addition, infectious causes of hepatic cysts range from liver abscesses caused by bacteria or entamoeba to echinococcal infections, which cause hydatid liver cysts.

Hepatic mucinous cystic neoplasms (MCNs, previously referred to as cystadenomas) present with an epithelium-lined solitary, large multiloculated cyst with mucinous content.^83^ It is important to rule out hydatid cysts through serological testing, as it looks similar to hepatic cystadenoma on imaging. In contrast to simple cysts, hepatic MCNs are more likely to be symptomatic and are at risk for growth and malignant transformation. In older adults, MCN can progress to cancer and are referred to as cystadenocarcinomas, which can be invasive and can even metastasize.^84^

Patients with autosomal-dominant polycystic liver disease, an inherited disorder closely related to polycystic kidney disease, also develop hepatic cysts.^85^ The cysts in autosomal-dominant polycystic liver disease may be similar to simple hepatic cysts in appearance; however, they can be differentiated from simple hepatic cysts by their pattern of distribution and growth. Autosomal-dominant polycystic liver disease cysts start as microcysts that show a steady progression in size over time. They show a bilobar, diffuse pattern of distribution throughout hepatic parenchyma and are more numerous in number than simple hepatic cysts.^86^ They may remain clinically silent until developing symptoms from secondary complications like intracystic infection, bleeding, rupture, or extrinsic pressure symptoms.

Caroli’s disease is a congenital disease with cystic biliary dilations that communicate with the biliary tract and usually presents between 5 and 20 years of age.^87^ Patients with Caroli’s disease usually become symptomatic due to recurrent bacterial infection of the cysts, which may present as recurring episodes of cholangitis.

Other rare causes of cystic lesions include biliary hamartomas, peribiliary cysts, hydatid cysts, intrahepatic biliary papillomatosis, and ciliated hepatic foregut cysts.^87^ Biliary hamartomas, also known as von Meyenburg complexes, are small, benign liver lesions resulting from bile duct malformations. These lesions are typically asymptomatic and discovered incidentally during imaging or surgery, often appearing as multiple small cysts scattered throughout the liver. Peribiliary cysts are usually located near the hepatic hilum and arise from peribiliary glands. These cysts are typically asymptomatic, although they can sometimes be associated with other liver diseases. Their presence is often detected incidentally through imaging studies. Hydatid cysts, caused by infection with the echinococcus parasite, are a more serious type of hepatic cyst. These cysts can cause significant morbidity due to their potential to grow large and cause pressure symptoms, rupture, or secondary infection.^88^ Intrahepatic biliary papillomatosis is a rare condition characterized by the proliferation of papillary tumors within the bile ducts.^89^ These tumors can lead to the formation of cystic lesions in the liver, are associated with a higher risk of malignancy, and may present with symptoms such as jaundice, abdominal pain, and cholangitis. Ciliated hepatic foregut cysts are rare congenital cysts lined with ciliated pseudostratified columnar epithelium.^90^ These cysts are typically asymptomatic and discovered incidentally, although they can occasionally cause symptoms if they become large or infected. These various types of benign hepatic cysts highlight the diversity of conditions that can affect the liver and underscore the importance of accurate diagnosis and appropriate management strategies tailored to the specific etiology of the cyst.

### Diagnostic strategies for hepatic cysts

Similar to other benign liver lesions, hepatic cysts are usually asymptomatic and maybe discovered incidentally on imaging done for other reasons. Once a liver cyst is identified, the diagnosis can be made by correlating the imaging characteristics of the cyst with the clinical and demographic characteristics of the differential diagnoses.^80^ An initial evaluation with ultrasound can help delineate whether the cyst is simple or complex. A simple cyst at ultrasound is anechoic with posterior acoustic enhancement, no color Doppler flow, no internal features such as septa, and a thin, imperceptible wall. A complex cyst has additional features, including the presence of septations or thickened walls. At CT, a simple cyst is seen as a nonenhancing, well-demarcated, water-attenuation lesion (<20 Hounsfield units) without associated perfusion differences in the surrounding parenchyma. On MRI, simple cysts appear hypointense on T1-weighted images, markedly hyperintense on T2-weighted images (ie, similar to cerebral spinal fluid), and nonenhancing after contrast administration. Multiphasic contrast examination is typically not necessary to confirm that a cyst is simple, although it may be helpful when the cyst is small.

MCN can be differentiated from simple cysts based on a combination of features, including thickened walls, wall nodularity, septations in the cyst, internal debris, and upstream biliary dilations. Biopsy or aspiration of MCN are generally nondiagnostic and are associated with peritoneal seeding and thus not recommended. Imaging features of hydatid cysts are distinctive on ultrasound and CT scans. On ultrasound, these cysts often display a characteristic “double-line sign,” indicative of the cyst wall, and may show “hydatid sand,” a collection of echinococcus scolices. CT imaging typically reveals well-defined cystic lesions that can contain multiple septations and daughter cysts within a larger cyst, providing a clear view of the complex, multivesicular structure typical of these infections.^91^ However, note that the imaging features vary with the lifecycle of the hydatid cyst and that a very early hydatid cyst may appear similar to a simple cyst. Biliary hamartomas, or von Meyenburg complexes, are typically small and multiple, and they appear as well-defined, nonenhancing lesions on both ultrasound and CT scans. MRI can further characterize these lesions, showing them as hypointense on T1 and hyperintense on T2-weighted images.^92^ Intrahepatic biliary papillomatosis presents as multiple cystic and papillary structures within the bile ducts, and MRI cholangiopancreatography can provide detailed images of these structures.

### Practical approach to the management of hepatic cysts

The management of hepatic cysts is tailored based on the presence of symptoms, the risk for cancer, and underlying etiology. The management of hepatic cysts has been reviewed extensively elsewhere^79^,^82^,^93^,^94^; here, we present a simplified practical approach to managing hepatic cysts. For asymptomatic simple cysts, a conservative management approach is typically appropriate. Once a diagnosis of a simple cyst is confirmed on imaging, no regular surveillance imaging is generally needed. Large simple cysts can be monitored with ultrasound if there is concern for growth. When simple cysts are symptomatic, they may need interventions to decrease the size and relieve the symptoms. Several therapeutic approaches are available for symptomatic large simple cysts. These include needle aspiration with the injection of a sclerosing agent, laparoscopic cyst deroofing, internal cyst drainage through cystojejunostomy, or even liver resection. When infectious cysts are suspected, the diagnosis can be confirmed through clinical evaluation, serologic tests (such as echinococcal serologies for hydatid cysts), and demographic correlation (considering factors like travel history to endemic areas for amebiasis). For hydatid cysts due to echinococcus, the treatment typically involves a combination of antiparasitic medications (eg, albendazole) and surgical intervention. The surgical approach may include cyst aspiration, drainage, or more definitive procedures like partial cystectomy or total cystectomy, depending on the cyst’s characteristics and location.

For MCNs, surgical resection is typically indicated due to the risk of malignancy. As mentioned above, biopsy or aspiration is generally nondiagnostic and carries a risk of peritoneal seeding, so definitive surgical removal is the preferred approach. In patients with polycystic liver disease, multiple cysts are present, and management focuses on symptom relief. Options include cyst fenestration or resection for dominant symptomatic cysts and, in severe or progressive cases, liver transplantation.

## OTHER RARE BENIGN LIVER TUMORS

Several other rare benign tumors can also arise within the liver. Even though these tumors are less frequently seen, it is important to recognize them due to their distinct clinical course. Below are some of the rare benign liver tumors.

Hepatic angiomyolipoma is a rare, benign neoplasm found predominantly in females.^95^ It usually occurs in the kidneys, but may occasionally occur in the liver and may be associated with tuberous sclerosis. It is composed of vasculature, smooth muscle cells, and adipose tissue in varying proportions. It may appear similar to HCC as they are both hypervascular, and both can contain macroscopic fat. Some angiomyolipomas are also “lipid-poor” and would be challenging to distinguish from other hypervascular lesions. Definitive diagnosis is made through immunohistochemical staining of resected lesions based on positive staining for homatropine methylbromide-45.^96^ Most hepatic angiomyolipomas can be monitored through surveillance, with resection recommended for uncertain biopsy results, high-risk features, symptom development, or aggressive growth.^96^ Hepatic hemangioendotheliomas are rare vascular tumors that can range from benign to low-grade malignant potential.^97^ On imaging, these tumors may appear as well-defined, hypervascular lesions.^98^ Symptoms can vary depending on the size and location of the tumor. While small, asymptomatic lesions can be monitored, larger or symptomatic tumors may require surgical resection. Hepatic schwannoma is a slow-growing benign tumor originating from Schwann cells, which usually occurs in the head, neck, and extremities, and may very rarely occur in the liver.^99^ It is composed of spindle cells in loose stroma, with signature Antoni A and Antoni B areas. Hepatic schwannoma is very difficult to diagnose preoperatively, and a definitive diagnosis is made through histopathological examination and immunohistochemical staining positive for the S100 and negative for c-kit and CD34 markers. It has a good prognosis with a low risk for recurrence after resection. Hepatic mesenchymal hamartoma is another rare tumor predominantly seen in children, usually in the first 2 years of life.^100^ It is composed of myxoid and fluid elements. It appears to have cystic and solid components on imaging and could be confused for a liver abscess. Hepatic mesenchymal hamartoma usually presents with abdominal mass and/or distension and has a good prognosis with excision.

## CONCLUSIONS

**FIGURE 7:**
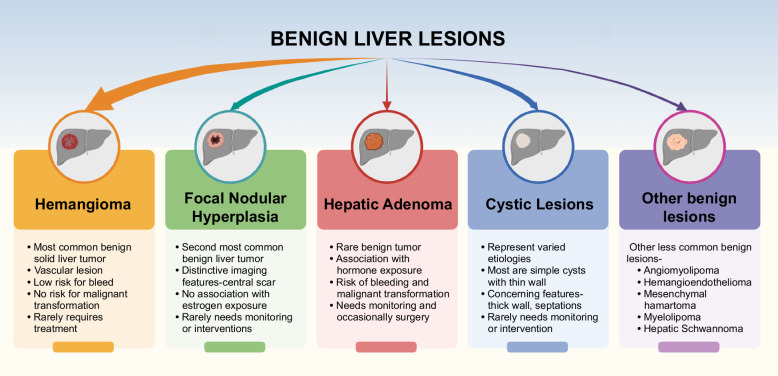
Overview of common benign liver lesions.

Benign liver lesions are common and can cause significant distress to patients due to their potential symptoms and the anxiety they provoke about malignancy. These lesions present a diagnostic dilemma for health care providers, necessitating a thorough clinical interview and physical examination to evaluate risk factors and guide further investigation. Advances in cross-sectional imaging, particularly MRI with hepatobiliary contrast agents, have greatly improved our ability to accurately diagnose these lesions. When imaging results are atypical or there is a concern for malignancy, tissue sampling should be considered. Among benign liver lesions, hepatic adenomas require special attention due to their risk of malignant transformation, bleeding, or significant growth. Management of hepatic adenomas should be tailored based on the subtype, incorporating considerations of the patient’s age, sex, and overall risk profile. Cystic lesions are generally benign; however, awareness of potential neoplasms like MCNs is crucial for appropriate management. Personalizing the management of benign liver lesions based on patient-specific factors, such as age, sex, risk factors, and patient preferences, ensures optimal care and improves outcomes. By staying informed about the characteristics and risks associated with various benign liver lesions, clinicians can navigate the diagnostic and therapeutic challenges these lesions present, ultimately enhancing patient care Figure 7.
